# *In Situ* and *in Vivo* Study of Nasal Absorption of Paeonol in Rats

**DOI:** 10.3390/ijms11124882

**Published:** 2010-11-26

**Authors:** Xiaolan Chen, Yang Lu, Shouying Du, Bing Xu, Shan Wang, Yongsong Zhai, Xiao Song, Pengyue Li

**Affiliations:** 1 School of Chinese Materia Medica, Beijing University of Chinese Medicine, No.6, Zhonghuan South Road, Wangjing, Chaoyang district, Beijing 100102, China; E-Mails: chenxiaolan76@126.com (X.C.); landocean88126.com (Y.L.); dushouying@263.net (S.D.); 2 Department of Chinese Pharmacy, Guiyang College of TCM, Guiyang, Guizhou 550002, China

**Keywords:** paeonol, nasal absorption, in situ, single pass perfusion technique, pharmacokinetics

## Abstract

The objective of this work was to study the *in situ* and *in vivo* nasal absorption of paeonol. A novel single pass *in situ* nasal perfusion technique was applied to examine the rate and extent of nasal absorption of paeonol by rats. Various experimental conditions, such as perfusion rate, pH, osmotic pressure and drug concentration, were investigated. The *in situ* experiments showed that the nasal absorption of paeonol was not dependent on drug concentration, and fitted a first order process. The absorption rate constant, *K*a, increased with an increase in perfusion speed. Paeonol was better absorbed in acidic solutions than in neutral or alkaline solutions. The value of *K*a was higher in a hypertonic environment than under isotonic or hypotonic conditions. *In vivo* studies of paeonol absorption were carried out in rats and the pharmacokinetics parameters of intranasal (i.n.) and intragastric (i.g.) administration were compared with intravenous (i.v.) administration. The bioavailabilities of paeonol were 52.37% and 15.81% for i.n. and i.g, respectively, while T_max_ values were 3.05 ± 1.46 min and 6.30 ± 0.70 min. MRT (Mean Residence Time) were 23.19 ± 6.46 min, 41.49 ± 2.96 min and 23.09 ± 5.88 min for i.n., i.g. and i.v. methods, respectively. The results demonstrate that paeonol could be absorbed promptly and thoroughly by i.n. administration in rats.

## 1. Introduction

Cortex Moutan, a kind of traditional Chinese medicine from *Paeonia Suffruticosa* Andr., has the function of clearing heat, absorbing clots, cooling and activating blood. Paeonol is an effective micro molecule phenolic compound isolated from Cortex Moutan. It is a kind of evaporable white needle crystal with low solubility. Recently, with the continuous development of research in natural medicines, the clinical application of paeonol has been further extended. Now, paeonol serves as a remarkable analgesic, anti-inflammatory and anti-atherosclerosis medicine and plays a protective role in cerebral ischemia [1]. In the clinic, paeonol is usually administered to patients orally or intramuscularly. Paeonol can be quickly absorbed by oral administration, but the bioavailability is relatively low compared with injection. Nevertheless, the high concentration of Tween-80 and ethanol used in injections can exert intense stimulations on muscles and blood vessels.

In the last decade, intranasal (i.n.) administration has drawn considerable interest since it provides a non-invasive method for bypassing first-pass effect and possibly the blood brain barrier [2–4]. The i.n. administration could be a hopeful substitution for injection, as drugs can be absorbed sufficiently and rapidly into the blood for systemic administration [5] and transported from the nasal cavity to the central nervous system [6]. About 40 substances have been reported to reach the brain via the direct nose-to-brain pathway [7]. Moreover, the i.n. route is safer than intravenous (i.v.) due to the barricade effect of the nasal mucosa. Based on these facts, great importance has been attached to the research on the nasal absorption of paeonol.

In this report, a novel single pass *in situ* nasal perfusion technique was applied to examine the rate and extent of nasal absorption of paeonol by rat. *In vivo* studies of paeonol were carried out in rats and the pharmacokinetics parameters of i.n. and i.g. were compared with that of i.v. administration.

## 2. Results and Discussion

### 2.1. *In Situ* Nasal Perfusion Experiments

Circulatory perfusion technology has been successfully applied to research the nasal absorption of drugs *in situ* over the past several years [8,9]. However, in our preliminary experiments, paeonol was found to be absorbed by the circulatory tubes made of common materials, such as PVA, rubber, silicone and low absorption silicone (Sani-Tech LA-60). The nasal absorption of paeonol cannot be researched by traditional circulation equipment. Therefore, a single pass perfusion device without soft tubes of peristaltic pump was innovated to study the nasal absorption of paeonol. This novel equipment and methods are also suited for the research of nasal absorption of drugs that are evaporable or absorbable by the tubes. Furthermore, it was found that water in the perfusate could be absorbed transnasally by rats. Hence, *K*a was calculated through gravimetry adjustment to get more precise and reliable results.

*Impact of perfusion speed on nasal absorption of paeonol:* Paeonol nasal solutions (100 μg/mL of paeonol) were prepared for *in situ* nasal perfusion (see Experimental section) at the speed of 0.2 mL/min, 0.3 mL/min and 0.4 mL/min, respectively. The results in Table 1 show that the absorption of paeonol could be affected by the perfusion speed.

*Impact of osmotic pressure on nasal absorption of paeonol:* Paeonol nasal solutions in purified water, normal saline (NS), or 1.8% NaCl solution (100 μg/mL of paeonol) were prepared for *in situ* nasal perfusion. It was shown that paeonol was absorbed better under conditions of high osmotic pressure than in neutral or low osmotic pressure conditions (as shown in Table 2).

*Impact of pH value on nasal absorption of paeonol:* Paeonol solutions of pH 4, pH 6.45 and pH 10 (100 μg/mL of paeonol; the pH was adjusted by buffer solution) were prepared according to the method of preparation of nasal solutions for in situ nasal perfusion. It was shown that paeonol was absorbed better in acidic conditions than in neutral or alkaline conditions (as shown in Table 3).

*Impact of concentration on nasal absorption of paeonol:* Paeonol solutions of 50, 100 and 200 μg/mL were prepared with NS (pH 6.45) according to the method of preparation of nasal solutions for *in situ* nasal perfusion in Section 3. Table 4 shows that absorption of paeonol had no concentration dependence. The absorption pattern appeared to follow the first order process, indicating that paeonol was transported across the nasal mucosa by passive diffusion.

### 2.2. *In Vivo* Studies

**Figure 1.**
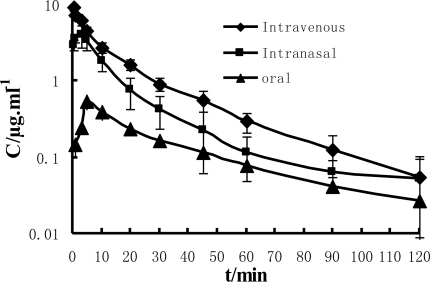
Concentration of paeonol in plasma (n = 6, mean ± S.D.) following a single i.v. (♦), i.n. (▪), or i.g. (▴) administration; calculated dosage of 4 mg·kg^−1^ of paeonol in rats.

The concentration-time curves resulting from administration of a single dose of paeonol by i.v., i.n. and i.g. in rats is shown in Figure 1. The i.v. as well as i.n. curve best fitted a two compartment open model. After i.v administration, the paeonol in plasma was eliminated rapidly. The plasma concentrations of paeonol decreased from 9.0 μg/mL to 0.9 μg/mL within 30 minutes. In the case of i.n. administration, the drug was absorbed rapidly. The C_max_ of paeonol reached about 4.0 μg/mL and decreased rapidly from 4.0 μg/mL to 0.5 μg/mL within 30 minutes. In the case of i.g. administration, the C_max_ of paeonol had reached approximately 0.4 μg/mL at the point of 6 min, and then decreased rapidly from 0.4 μg/mL to 0.05 μg/mL within 30 minutes. The main pharmacokinetic parameters of the plasma after i.v., i.n. and i.g. administration are shown in Table 5. The Kel values were 0.082 min^−1^, 0.14 min^−1^ and 0.054 min^−1^ for i.v., i.n. and p.o., respectively. MRT for injection, nasal and oral solutions were 23.09, 23.19 and 41.49 min, respectively while AUC (Area Under the Concentration-Time Curve) values were 111.62, 58.45 and 17.65 μg/mL·min. Our present results demonstrated that after the i.n. administration, paeonol could be absorbed rapidly and the bioavailability of paeonol was relatively high.

## 3. Experimental

### 3.1. Chemicals, Reagents and Animals

Paeonol was obtained from the National Institute for the Control of Pharmaceutical and Biological Products (NICPBP, Beijing, P.R. China). Acetonitrile, methanol and water were of HPLC grade (Qualigens, China) and all other reagents were of analytical grade. Male Sprague Dawley (SD) rats (230–250 g) were obtained from WeiTong biotechnology Inc. (Beijing, China), and were kept in a controlled-environment breeding room (temperature: 22 ± 1 °C, humidity: 60 ± 5%, 12-h dark/light), with free access to common food and water in the first week. All experimental procedures were conducted in accordance with the European Union guidelines for the use of experimental animals and approved by the Beijing University of Chinese Medicine Committee on Animal Care and Use.

### 3.2. Instrumentation

The LC used was an SHIMADZU 10AD series LC (SHIMADZU LC- HP Inc. Japan,) with a binary pump, on-line degasser, and a thermostated autosampler. The separation was performed on a Prevail C_18_ column (250 × 4.6 mm, 5 μm, Dikma Technology Company, China).

### 3.3. Chromatographic Conditions

The mobile phases were methanol and water (60:40, v/v), and the signal was monitored at 274 nm. The flow rate was maintained at 1.0 mL/min. All the samples were centrifuged at 12,000 rpm for 10 minutes before determination. There was a good linearity between A and C (*A* = 5354*C* + 1783.8 *r* = 0.9999). The validation parameters of precision (CV less than 1%) were acceptable and the lower limit of quantitation was 5.056 μg/mL.

### 3.4. Sample Preparation

#### 3.4.1. *In Situ* Nasal Perfusion Experiments

##### 3.4.1.1. Preparation of Nasal Solutions

0.4% (v/v) of Tween-80 was dissolved in physiological saline and used as the solvent for drugs. Paeonol was dissolved according to the ratio of desired concentration for *in situ* nasal single pass perfusion experiments.

##### 3.4.1.2. Methods of *in Situ* Nasal Single Pass Perfusion

**Figure 2.**
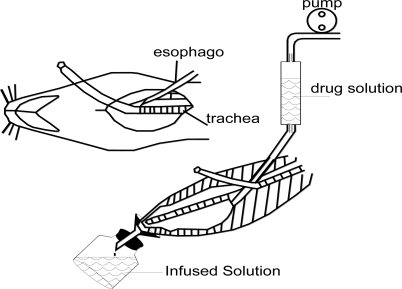
Diagram of *in situ* nasal single pass perfusion.

The absorption studies were carried out using the novel *in situ* nasal single pass perfusion technique (as shown in Figure 2). Rats were anesthetized by intraperitoneal injections of urethane (1.2 g/kg body weight). An incision was made in the rat neck. The trachea was cannulated with a polyethylene tube to allow breathing while another tube was inserted through the esophagus into the posterior part of the nasal cavity. The nasopalatine duct was closed with cyanoacrylate glue to prevent the drainage of solution from the nasal cavity to the mouth. The tube inserted into the esophagus was connected to a storage bottle containing 20 mL drug solution. The whole passage was sealed in order to avoid the volatilization of paeonol.

The drug solution was infused into the nasal cavity by the pressure generated from a peristaltic pump at a certain speed. After running through the nasal cavity, the perfusate was collected into a receiving bottle every 10 minutes (10, 20, 30, 40, 50 and 60 min as scheduled). The bottle with perfusate was weighed immediately when it was substituted. Then, a certain amount of perfusate was sampled, filtrated and analyzed by HPLC to determine the content of paeonol. The first-order rate constant of the absorption of paeonol, *Ka,* was estimated by Equation (1).

(1) $$\text{Ka}=(1-\frac{\rho_{\text{out}}}{\rho_{\text{in}}}•\frac{V_{\text{out}}}{V_{\text{in}}})•\frac{v}{V_{\text{nose}}}$$

where V_in_ corresponds to volume of drug solution perfused in 10 min; V_out_ is the volume of outflow perfusate received in 10 min (V_out_ ≈ W_out_ for density of perfusate approximately equal to 1 g/mL, W_out_ is the weight of outflow perfusate received in 10 min); *ν* is the speed of perfusion; ρ_in_ and ρ_out_ are concentrations of paeonol (mg·mL^−1^) of buffers in the entrance and exit, respectively; V_nose_ is the volume of the rat nasal cavity (calculated by the volume of drug solution contained in it). The *in situ Ka* of the nasal absorption groups were compared by one-way ANOVA at the 0.05 significance level, and the tests for statistical differences were paired *t*-tests. (Statistics Analysis System 8.0).

##### 3.4.2. *In Vivo* Experiments

Fifteen Male SD rats, weighing 230∼250 g, were randomly assigned to three groups. All animals were fasted for 12 hours prior to the experiments and anesthetized with an intraperitoneal injection of urethane (1.2 g/kg body weight). About 0.5 mL of injection sample (at a single dose of 4 mg/kg (for paeonol)) was either injected via tail vein or administered via nostril by a modified micro-injector as a 50 μL i.n. solution. For oral administration, 1 mL of solution was intragastriced at a single dose of 40 mg/kg (for paeonol), the paeonol was dissolved by the complex solvent (88% physiological saline, 10% ethanol and 2% Tween-80). 0.25 mL of blood was collected from the left carotid artery at 0.5, 1, 3, 5, 10, 20, 30, 60, 90, 120 min after the drug administration. Blood samples were placed into heparinized tubes. After centrifugation, the plasma obtained was stored at −20 °C until determination. An aliquot of 100 μL of plasma sample was placed into a centrifuge tube and 300 μL acetonitrile was added. After 1 min in a vortex, the mixture was centrifuged at 12,000 rpm for 10 min. 20 μL acetonitrile solution was injected into the HPLC system. The HPLC conditions were listed above. It could be seen that there was a good linearity between C and A (C = 0.00004A - 0.1215, *r* = 0.9999). The validation parameters of precision (CV less than 5%) and accuracy (recovery of ± 20%) were acceptable and the lower limit of quantitation was 0.051 μg/mL. The pharmacokinetic parameters associated to each animal were estimated using Kinetica 4.4 software. The *in vivo* pharmacokinetic parameters of the i.n. and i.v. and i.g. groups were compared by one-way ANOVA at the 0.05 significance level, and the statistical difference was analyzed by paired t-tests (Statistics Analysis System 8.0).

## 4. Conclusions

Both in modern and traditional pharmaceutics, nasal drug delivery (NDD) is considered to be an effective and promising method for drug administration. This route is beneficial for drugs which can be absorbed sufficiently and rapidly into the systemic circulation and can be transported adequately to the brain as mentioned above. Paeonol is often administered either by injection or orally. However, the bioavailability of paeonol is low through oral administration, and the administration by the i.v. route is inconvenient. Therefore, our research on the nasal absorption of paeonol is of great values.

Circulatory perfusion technology has been successfully applied to research the *in situ* nasal absorption of drugs. However, paeonol was found to be absorbed by the circulatory tubes. We took a novel single pass perfusion device to study the nasal absorption of paeonol. From investigations of the effects of perfusion speed, osmotic pressure, pH value and drug concentration on the *in situ* nasal absorption of paeonol, it was shown that the *K*a value would increase with increasing perfusion speed. However, a high perfusion speed may cause injury of the nasal mucosa so that the drug absorption is accelerated and the measured value may differ from the reality. Therefore, the perfusion speed of 0.2 mL/min was adopted in subsequent experiments. Under hypertonic conditions, the *K*a was greater than that in isotonic and hypotonic environments (*P* < 0.01). This is possibly due to the cells on the nasal mucosa shrinking in hypertonic solutions, leading to the opening up of intercellular space. The pH value of the administering solution also influenced the nasal absorption of paeonol. Research indicated that paeonol was better absorbed under acidic conditions than in neutral or alkaline conditions, possibly because more paeonol dissociated in neutral or alkaline conditions than in acidic conditions. Since drug concentration had no effect on *K*a, the absorption of paeonol could be deemed as fitting for the first order process.

Pharmacokinetic studies of paeonol through i.n. administration routes in rats revealed that paeonol was rapidly absorbed and quickly eliminated. The C_max_ of paeonol by i.n. administration was achieved in three minutes. Moreover, after 30 min, the blood concentration of paeonol was lowered to 10% of the C_max_. In addition, the relative bioavailability of paeonol by i.n. administration was 52.37%—about three-times more than the bioavailability of i.g. administration, which was 15.81%.

The results of our study, both *in situ* and in *vivo,* indicated that paeonol can be absorbed by the nasal route. Owing to the safety, convenience and cheapness of nasal administration compared with injection treatment, developing new quick-action preparations of paeonol based on NDD technologies is valuable, encouraging and worthy of further studies.

## Figures and Tables

**Table 1.** *K*a of paeonol nasal absorption with different perfusion speeds (*n* = 5, mean ± S.D.)

**Perfusion Speed (mL/min)**	***K*a·10^−2^ min^−1^**
0.2	88.59 ± 6.47
0.3	138.57 ± 9.97^**^
0.4	142.66 ± 11.95^**^

** *P* < 0.01 *vs.* 0.2.

**Table 2.** *K*a of paeonol nasal absorption with different osmotic pressures (*n* = 5, mean ± S.D.).

**Solution**	***K*a·10^−2^ min^−1^**
Pure Water	91.62 ± 9.26
NS	88.59 ± 6.47
1.8% NaCl	109.47 ± 9.54^**^

** *P* < 0.01 *vs.* 0.9% NaCl.

**Table 3.** *K*a of paeonol nasal absorption with different pH (*n* = 5, mean ± S.D.).

**pH Value**	***K*a·10^−2^ min^−1^**
4.01	98.97 ± 11.68^**^
6.45	88.59 ± 6.47
10.0	81.87 ± 17.91

** *P* < 0.01 *vs.* pH 6.45

**Table 4.** *K*a of paeonol nasal absorption with different drug concentrations (*n* = 5, mean ± S.D.).

**Drug Concentration (μg/mL)**	***K*a·10^−2^ min^−1^**
50	88.91 ± 3.66
100	88.59 ± 6.47
200	93.76 ± 4.88

**Table 5.** Main pharmacokinetic parameters of plasma after i.v. and i.n. and i.g. administration of paeonol in rats (*n* = 5, mean ± S.D.).

**Parameter**	**Unit**	**i.v.**	**i.n.**	**i.g.**
ka	Min^−1^	-	0.73 ± 0.19	0. 43 ± 0.34
A	μg·mL^−1^	5.51 ± 1.84	6.57 ± 1.68	6.83 ± 3.86
α		0.14 ± 0.12	0.29 ± 0.14	0. 15 ± 0.03
B	μg·mL^−1^	4.04 ± 1.75	1.17 ± 0.48	2.25 ± 0.66
β		0.09 ± 0.11	0.037 ± 0.014	0.018 ± 0.002
AUC	μg/mL·min	111.62 ± 14.45	58.45 ± 12.88**	17.65 ± 3.44**
MRT	min	23.09 ± 5.88	23.19 ± 6.46	41.49 ± 2.96**
Cmax	μg·mL^−1^	8.96 ± 0.84	4.50 ± 1.53**	0.49 ± 0.10**
Tmax	min	-	3.05 ± 1.46	6.29 ± 0.70
Kel	min^−1^	0.08 ± 0.015	0.14 ± 0.044**	0.053 ± 0.017
K12	min^−1^	0.065 ± 0.042	0.11 ± 0.078**	0.057 ± 0.025
K21	min^−1^	0.087 ± 0.058	0.077 ± 0.035	0.05 ± 0.02**
T1/2 Ka	min	-	0.87 ± 0.57	2.44 ± 1.33
T1/2α	min	8.99 ± 6.99	3.16 ± 2.06**	4.97 ± 1.12**
T1/2β	min	21.28 ± 20.41	23.769 ± 16.86*	39.026 ± 5.10**

* *P* < 0.05 *vs.* i.v.,

** *P* < 0.01 *vs.* i.v.
